# Human behavioral complexity peaks at age 25

**DOI:** 10.1371/journal.pcbi.1005408

**Published:** 2017-04-13

**Authors:** Nicolas Gauvrit, Hector Zenil, Fernando Soler-Toscano, Jean-Paul Delahaye, Peter Brugger

**Affiliations:** 1 Algorithmic Nature Group, Laboratoire de Recherche Scientifique LABORES For the Natural and Digital Sciences, Paris, France; 2 Human and Artificial Cognition Lab, EPHE, Paris, France; 3 Department of Computer Science, University of Oxford, Oxford, United Kingdom; 4 Information Dynamics Lab, Unit of Computational Medicine, Department of Medicine Solna, Centre for Molecular Medicine and Science for Life Laboratory (SciLifeLab), Karolinska Institute, Stockholm, Sweden; 5 Grupo de Lógica, Lenguaje e Información. Universidad de Sevilla, Sevilla, Spain; 6 Centre de Recherche en Informatique, Signal et Automatique de Lille (CRISTAL), UMR CNRS 9189, University of Lille 1, Lille, France; 7 Department of Neurology, Neuropsychology Unit, University Hospital, Zurich, Switzerland; Instituto Superior Técnico, Univesidade de Lisboa, PORTUGAL

## Abstract

Random Item Generation tasks (RIG) are commonly used to assess high cognitive abilities such as inhibition or sustained attention. They also draw upon our approximate sense of complexity. A detrimental effect of aging on pseudo-random productions has been demonstrated for some tasks, but little is as yet known about the developmental curve of cognitive complexity over the lifespan. We investigate the complexity trajectory across the lifespan of human responses to five common RIG tasks, using a large sample (*n* = 3429). Our main finding is that the developmental curve of the estimated algorithmic complexity of responses is similar to what may be expected of a measure of higher cognitive abilities, with a performance peak around 25 and a decline starting around 60, suggesting that RIG tasks yield good estimates of such cognitive abilities. Our study illustrates that very short strings of, i.e., 10 items, are sufficient to have their complexity reliably estimated and to allow the documentation of an age-dependent decline in the approximate sense of complexity.

## Author summary

It has been unclear how this ability evolves over a person’s lifetime and it had not been possible to be assessed with previous classical tools for statistical randomness. To better understand how age impacts behavior, we have assessed more than 3,400 people aged 4 to 91 years old. Each participant performed a series of online tasks that assessed their ability to behave randomly. The five tasks included listing the hypothetical results of a series of 12 coin flips so that they would “look random to somebody else,” guessing which card would appear when selected from a randomly shuffled deck, and listing the hypothetical results of 10 rolls of a die. We analyzed the participants’ choices according to their algorithmic randomness, which is based on the idea that patterns that are more random are harder to encode in a short computer program. After controlling for characteristics such as gender, language, and education. We have found that age was the only factor that affected the ability to behave randomly. This ability peaked at age 25, on average, and declined from then on. We also demonstrate that a relatively short list of choices, say 10 hypothetical coin flips, can be used to reliably gauge randomness of human behavior. A similar approach could be then used to study potential connections between the ability to behave randomly, cognitive decline, neurodegenerative diseases and abilities such as human creativity.

## Introduction

Knowledge forming the content of several academic fields, including mathematics, follows from precocious core knowledge [1–3], and then follows a specific developmental course along the lifespan [4]. Numerosity (our approximate implicit sense of quantity) has been a privileged target of recent research, because numbers form one of the main pillars of elementary mathematical knowledge [5], but the study of randomness perception and statistical reasoning has also yielded striking results in the field of probability: adults with no formal education [6] as well as 8 to 12 month-old children [7, 8] have the wherewithal for simple implicit probabilistic reasoning. One of the toughest problems when it comes to Bayesian reasoning, however, is the detection of randomness, i.e., the ability to decide whether an observed sequence of events originates from a random source as opposed to produced by a deterministic origin [9].

Formally, the algorithmic (Kolmogorov-Chaitin) complexity of a string is the length of the shortest program that, running on a universal Turing machine (an abstract general-purpose computer), produces the string and halts. The algorithmic complexity of a string is a measure of how likely it is to have been produced deterministically by a computer program rather than by chance. In this way, a random string is a string that cannot be compressed by any means, neither statistically or algorithmically, that is a string for which no computer program shorter than the string itself exists. Humans, adults and infants [10, 11], have a keen ability to detect structure, both of statistic and algorithmic nature (e.g. 0101… and 1234…) that only algorithmic complexity can intrinsically capture (as opposed to e.g. entropy rate).

Within the field of study devoted to our sense of complexity, the task of randomly arranging a set of alternatives is of special interest, as it poses almost insurmountable problems to any cognitive system. The complexity of a subject-produced pseudorandom sequence may serve as a direct measure of cognitive functioning, one that is surprisingly resistant to practice effects [12] and largely independent of the kind of alternatives to be randomized, e.g., dots [13], digits [14], words [15], tones [16] or heads-or-tails [17]. Although random item generation (RIG) tasks usually demand vocalization of selections, motor versions have comparable validity and reliability [18, 19]. RIG tasks are believed to tap our approximate sense of complexity (ASC), while also drawing heavily on focused attention, sustained attention, updating and inhibition [20, 21]. Indeed to produce a random sequence of symbols, one has to avoid any routine and inhibit prepotent responses. The ability to inhibit such responses is a sign of efficient cognitive processing, notably a flexibility assumed to be mediated by the prefrontal cortex.

Instructions may require responding at various speeds [22], or else the generation of responses may be unpaced [27]. Participants are sometimes asked to guess a forthcoming symbol in a series (“implicit randomization”, [51]), or vaguely instructed to “create a random-looking string” [23]. The consensus is that, beyond their diversity, all RIG tasks rely heavily on an ASC, akin to a probabilistic core knowledge [24, 25].

Theoretical accounts of the reasons why RIG tasks are relevant tests of prefrontal functions are profuse, but pieces of experimental evidence are sparse. Sparse empirical factors indirectly validate the status of RIG tasks as measures of controlled processing, such as the detrimental effect of cognitive load or sleep deprivation [26] or the fact that they have proved useful in the monitoring of several neuropsychological disorders [27–31].

As a rule, the development of cognitive abilities across the lifespan follows an inverse U-shaped curve, with differences in the age at which the peak is reached [4, 32]. The decrease rate following the peak also differs from one case to another, moving between two extremes. “Fluid” components tend to decrease at a steady pace until stabilization, while “crystalized” components tend to remain high after the peak, significantly decreasing only in or beyond the 60s [33]. Other evolutions may be thought of as a combination of these two extremes.

Two studies have addressed the evolution of complexity in adulthood, but with limited age ranges and, more importantly, limited ‘complexity’ measures. The first [22] compared young and older adults’ responses and found a slight decrease in several indices of randomness. The second [15] found a detrimental effect of aging on inhibition processes, but also an increase of the cycling bias (a tendency to postpone the re-use of an item until all possible items have been used once), which tends to make the participants’ productions more uniform. In both studies, authors used controversial indices of complexity that only capture particular statistical aspects, such as repetition rate or first-order entropy. Such measures have proved some usefulness in gauging the diversity and type of long sequences (with e.g., thousands of data points) such as those appearing in the study of physiological complexity in [34–36], but are inadequate when in comes to short strings (e.g., of less than a few tens of symbols), such as the strings typically examined in the study of behavioral complexity. Moreover, such indexes are only capable of detecting statistical properties. Authors have called upon algorithmic complexity to overcome these difficulties [37, 38]. However, because algorithmic complexity is uncomputable, it was believed to have no practical interest or application. In the last years, however, methods were introduced related to algorithmic complexity that are particularly suitable for short strings [39, 40], and native *n*-dimensional data [41]. These methods are based on a massive computation to find short computer programs producing short strings and have been made publicly available [42] and have been successfully applied in a range of different applications [41, 43, 44].

The main objective of the present study is to provide the first fine-grained description of the evolution over the lifespan of the (algorithmic) complexity of human pseudo-random productions. Secondary objectives are to demonstrate that, across a variety of different tasks of random generation, the novel measure of behavioral complexity does not rely on the collection of tediously long response sequences as hitherto required. The playful instructions to produce brief response sequences by randomizing a given set of alternatives are suitable for children and elderly people alike, can be applied in work with various patient groups and are convenient for individual testing as well as Internet-based data collection.

Participants with ages ranging from 4 to 91 performed a series of RIG tasks online. Completion time (CT) serves as an index of speed in a repeated multiple choice framework. An estimate of the algorithmic complexity of (normalized) responses was used to assess randomization performance (e.g., response quality). The testing hypothesis is that the different RIG tasks are correlated, since they all rely on similar core cognitive mechanisms, despite their differences. To ensure a broad range of RIG measurements, five different RIG tasks were selected from the most commonly used in psychology.

The experiment is some sort of reversed Turing test where humans are asked to produce configurations of high algorithmic randomness that are then compared to the occurrence of what computers can produce by chance according to the theory of algorithmic probability [39, 40].

## Methods

### Ethics statement

This study was approved by the University of Zurich Institutional Review Board (Req00583).

The five tasks used, described in Table 1, are purposely different in ways that may affect the precise cognitive ability that they estimate. For instance, some tasks draw on short-term memory because participants cannot see their previous choices (e.g., “pointing to circles”), whereas in other tasks memory requirements are negligible, because the participant’s previous choices remain visible (“rolling a die”). Completion times across the various tasks showed a satisfactory correlation (Cohen’s *α* = .79), suggesting that participants did not systematically differ in the cognitive effort they devoted to the different tasks. Any difference between task-related complexities is thus unlikely to be attributable to differences in time-on-task.

**Table 1:** Description of the 5 RIG tasks used in the experiment. Order was fixed across participants.

Task	Description
Tossing a coin	Participants had to create a series of 12 head-or-tails that would “look random to somebody else” by clicking on one of the two sides of a coin appearing on the screen. The resulting series was not visible on the screen (the participant could only see the last choice made).
Guessing a card	Participants had to select one of 5 types of cards (Zener cards; see e.g. [45]), ten times. In contrast to the other tasks, they were not asked to make the result look random. Instead, they were asked to guess which card will appear after a random shuffle.
Rolling a die	Participants had to generate a string of 10 numbers between 1 and 6, as random as possible (“the kind of sequence you’d get if you really rolled a die”). In contrast to the preceding cases, they could here see all previous choices, but could not change any of them.
Pointing to circles	Participants had to point 10 times at one out of 9 circles displayed simultaneously on the screen. They could not see their previous choices. This task is an adaptation of the classical Mittenecker pointing test [13].
Filling a grid	Participants had to blacken cells in a 3x3 grid such that the result would look randomly patterned, starting from a white grid. In contrast to the other tasks, they could see their choice and click as many times as they wished. Clicking on a white cell made it black, and vice versa.

**Fig 1:**
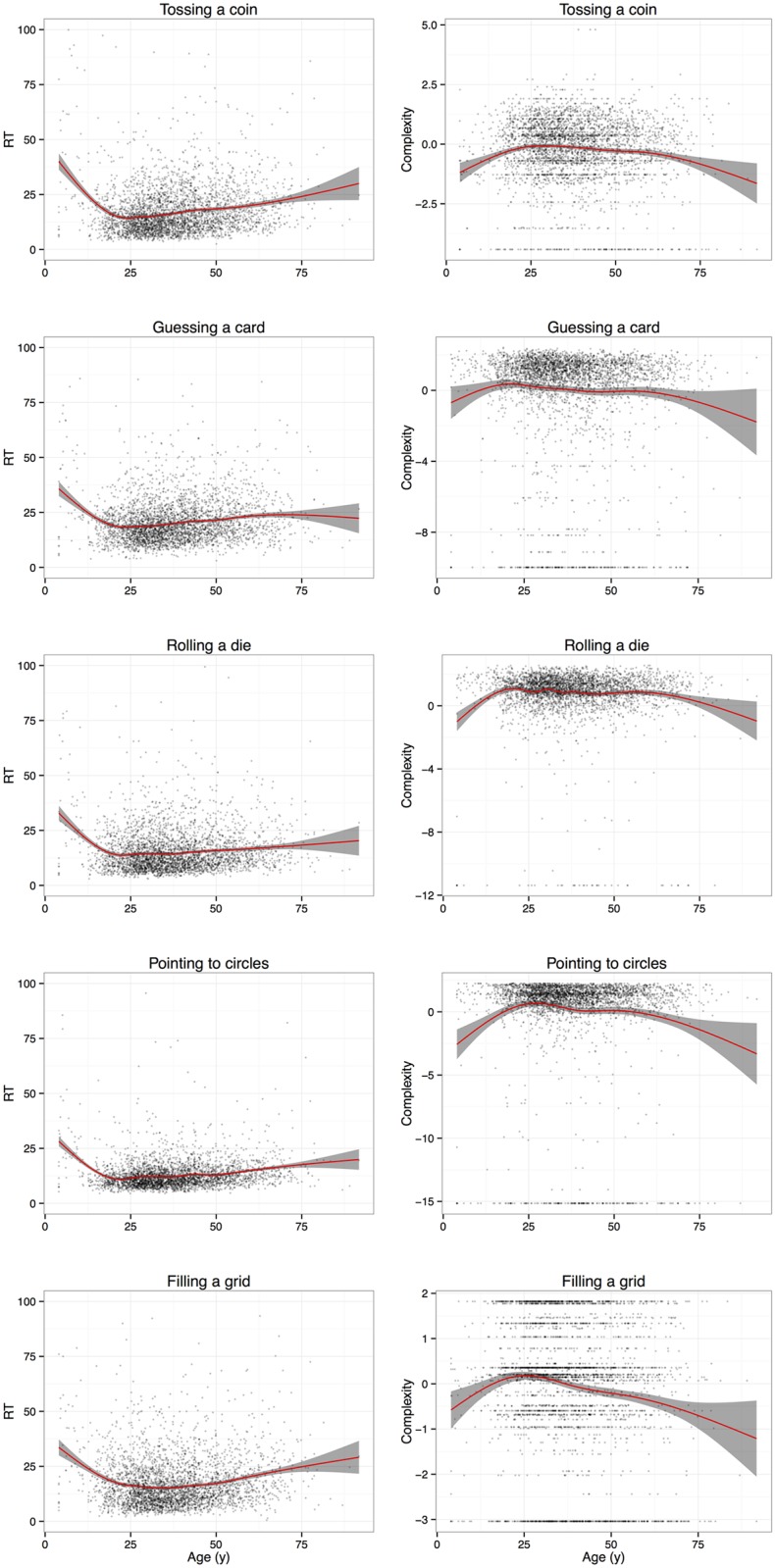
Developmental curves of completion time and complexity, split by task, with trend curves and 95% confidence regions (shaded).

Complexities were weakly to moderately positively correlated across the different tasks (Cohen’s *α* = .45), mostly as a consequence of the “filling the grid” task being almost uncorrelated with the other tasks (for more details, see *SI Principal Component Analysis*). Despite this moderate link, however, all trajectories showed a similar pattern across the lifespan, with a peak around 25, a slow, steady decline between 25 and 60, followed by accelerated decline after 60 as shown in Fig 1.

The hypothesis to test that the different tasks are positively related to each other was partially supported by the data, especially in view of the results obtained on the “filling the grid” task. At the same time, CTs showed correlations supporting the testing hypothesis together with the developmental complexity curves in agreement pointing in the same direction. This suggests that all tasks do tap into our ASC as well as into other cognitive components with similar developmental trajectories, but that different tasks actually require different supplementary abilities or else they weight the components of these abilities differently.

The “filling the grid” task appeared unique in that it was loosely correlated with all the other tasks. The fact that it required binary responses cannot account for this lack of association, since the “tossing a coin” task yielded results uncorrelated with the “filling the grid” responses. Bi-dimensionality could possibly have had an effect, but the “pointing to circles” task was also unrelated to the grid task. On the other hand, one factor distinguished the grid task from all others in the set: the option offered to the participants to change their previous choices as many times as they wished. For that reason, the grid task may in fact have relied more on randomness perception, and less on inhibition and randomness production heuristics. Indeed, participants could change the grid until they felt it was random, relying more on their ASC than on any high order cognitive ability serving output structure. This hypothesis is supported by the fact that participants did indeed change their minds. There were only 9 cells (that could turn white or black) on the grids and participants’ end responses had a mean of 4.08 (*SD* = 1.8) selected (black) cells thus generally favoring whiter configurations (possibly as a result of the all-white initial configuration). However, the number of clicks used by participants during this task was far larger (*M* = 10.16, *SD* = 9.86), with values ranging from 5 to 134 (this latter trying almost a fifth of all possible configurations). Thus the option to change previous choices in a RIG task may have been an important factor, and should accordingly be considered a novel variable in future explorations of randomization tasks (and balanced with an all-black configuration). In this view, the “filling the grid” task would reflect our ASC in a more reliable fashion than other tasks, while being less dependent on inhibition processes.

### Limitations

The present findings are based on data collected online. One could argue that this might bias the sample toward participants more comfortable with technology. Although direct control over a participant’s behaviour online is certainly limited, as compared to the laboratory environment, there is an increasing number of studies demonstrating the convergence of laboratory-based and web-based data collection [4]. This is the case in very particular procedural situations, such as lateralized [46] or very brief [47] stimulation, presentation and the measurement of reaction times [48], and it also holds for the assessment of cognitive changes across the lifespan [49]. Compared to these special situations, our research procedure was simple, the tasks were entertaining, and response times did not have to be collected with millisecond precision [50]. We thus think that any disadvantages of online-testing have been more than compensated for by the advantages of enrolling a large number of participants across a wide age range.

### Modulating factors

To investigate possible modulating factors (besides age), we used general linear models with complexity and CT as DV, and age (squared), sex, education, field and paranormal beliefs as IV.

The variable *Sex* was chosen in order to test in a large sample whether the absence of differences in laboratory RIG experiments could be replicated in an online test. Similarly, *Education* was important to test given previous claims in the RIG literature that human randomization quality may be independent of educational level [51]. Paradoxically, participants with a scientific background may perform worse at producing random sequences, thanks to a common belief among them that the occurrence of any string is as statistically likely as any other (a bias deriving from their knowledge of classical probability theory), which further justifies controlling for *Field of education*, simplified as humanities v. science. Finally, the variable Paranormal Belief was included as it has been related to RIG performance in previous studies [52].

The variables *field* and *paranormal belief* were, however, only asked in a subset of the 3313 participants that were above the age of 15 and we ignored the responses of younger participants as they were not considered to have a differentiated education background nor a fixed belief concerning paranormality. The analysis was performed on a task-wise basis. As we report, neither field or education level had no significant effect on any of the complexity or CT scores.

### Experiment

A sample of 3429 participants took part in the experiment (age range: 4–91y, M = 37.72, SD = 13.38). Participants were recruited through social networks, radio broadcasts and journal calls during a 10 month period. Basic demographic characteristics of the sample are displayed in Table 2, the experiment is to this date still available online for people to test (URL available in the next section). Each of the five (self-paced) RIG tasks consisted in the production, as fast and accurately as possible, of a short (with length range 9–12) pseudo-random series of symbols, with various numbers of possible symbols and variations among other instructional features (Table 1).

**Table 2:** Sample descriptive statistics (*n*).

Sex	Male	2333
	Female	1085
	Unknown	11
Mother tongue	English	274
	French	1448
	German	1303
	Spanish	220
	Other	184
Education level	Kindergarten or below	38
	Primary school	83
	Secondary school	387
	High School	621
	Undergraduate	347
	Graduate	1364
	Post graduate	538
	Unknown	51
Field	Humanities	609
	Science	1684
	Other	550
	Irrelevant	586

### Procedure

A specific web application was designed to implement the experiment online. Participants freely logged on to the website (http://complexitycalculator.com/hrng/). The experiment was available in English, French, German, and Spanish (all translated by native speakers for each language). In the case of young children as yet unable to read or use a computer, an adult was instructed to read the instructions out loud, make sure they were understood, and enter the child’s responses without giving any advice or feedback. Participants were informed that they would be taking part in an experiment on randomness. They then performed a series of tasks (all participants performed the tasks in this order, see *SI* for screen shots) before entering demographic information such as sex, age, mother tongue, educational level, and main field of education (science, humanities, or other) if relevant. Before each task, participants (or a parent, in the case of youngsters) read the specific instructions of the task and only press “start” key after full agreement that they have understood the requirements of the task. Only then, that action initiated the measurement of the completion time (CT) for each task, which was recorded alongside the responses. Practice trials were not allowed in order to minimize boredom effects leading to drop-out rates and bias and to maximize spontaneity.

One last item served as an index of paranormal beliefs and was included since probabilistic reasoning is among the factors associated with the formation of such beliefs [53, 54]. Participants had to rate on a 6-point Likert scale how well the following statement applied to them: “Some ‘coincidences’ I have experienced can best be explained by extrasensory perception or similar paranormal forces.”

### Measures

For each task, CT (in seconds) was recorded. The sum of CTs (total CT) was also used in the analyses. An estimate of the algorithmic complexity of participants’ responses was computed using the acss function included in the freely publicly available acss R-package [42] that implements the complexity methods used in this project. Complexities were then normalized, using the mean and standard deviation of all possible strings with the given length and number of possible symbols, so that a complexity of 0 corresponds to the mean complexity of all possible strings. For each participant, the mean normalized complexity (averaged over the five tasks) was also computed, serving as a global measure of complexity.

## Results and discussion

Sex had no effect on any of the complexity scores, but a significant one on two CT scores, with male participants performing faster in the first two tasks: “tossing a coin” (*p* = 6.26 × 10^−10^, $\eta_{p}^{2}=.012$) and “guessing a card” (*p* = 2.3 × 10^−10^, $\eta_{p}^{2}=.012$). A general linear model analysis of the total CT scores as a function of sex, age (squared), field, education level and paranormal belief was performed on the same subset and revealed a strongly significant effect of sex (*p* = 9.41 × 10^−8^, $\eta_{p}^{2}=.009$), with male participants performing faster than female participants. A simpler model, including only sex and age (squared) as IV, still showed an effect of sex (*p* = 6.35 × 10^−13^, $\eta_{p}^{2}=.016$), with male participants needing less time. The sex difference in CT, mostly appearing in adulthood (during the 60s; *SI, Fig. 4*), was in line with previous findings that in adults, choice CT is lower in men than in women [55].

Paranormal belief scores were unrelated to CTs for all tasks. However, they were negatively linked with the complexity score in the “filling a grid” task (*p* = .0006, $\eta_{p}^{2}=.004$), though not with any other task.

Paranormal beliefs have been previously reported to be negatively linked to various RIG performances [52, 53]. Our results replicated these findings but only on the “filling a grid” task. One possible explanation is that the grid task actually measures randomness perception rather than randomness production, and that a paranormal bent is more strongly linked with a biased perception of randomness than with a set of biased procedures used by participants to mimic chance. This hypothesis is supported by the finding that believers in extrasensory perception are more prone to see “meaningful” patterns in random dot displays [52]. Another complementary hypothesis is that the type of biases linked to beliefs in the paranormal only usually appear over the long haul, and are here preempted by the fact that we asked participants to produce short sequences (of 12 items at most). Indeed, when it comes to investigating the effects of paranormal belief on pure randomness production, rather long strings are needed, as the critical measure is the number of repetitions produced [53, 56].

To get a better sense of the effect of paranormal belief, we performed a general linear model analysis of the mean complexity score as a function of sex, age (squared), field, education level and paranormal belief. For this analysis, we again used the subset of 3,313 participants over the age of 15. Paranormal belief no longer had an effect.

### Mean complexity and total CT trajectories

**Fig 2:**
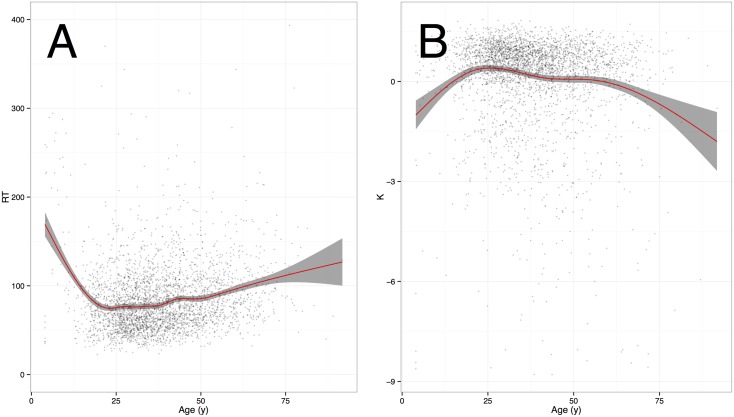
(A) Total completion time (CT) and (B) mean complexity as a function of age, with trend curve and 95% confidence region (shaded area).

Our main objective was to describe the evolution over the lifespan of mean complexity, which is achieved here using an approximation of algorithmic complexity for short strings (but see *SI Entropy* for a discussion of entropy (under)performance). Following Craik and Bialystok’s [33] view, the developmental curve of complexity found in Fig 2, suggests that RIG tasks measure a combination of fluid mechanics (reflected in a dramatic performance drop with aging) and more crystallized processes (represented by a stable performance from 25 to 65 years of age). This trajectory indirectly confirms a previous hypothesis [15]: attention and inhibition decrease in adulthood, but an increased sense of complexity based on crystallized efficient heuristics counters the overall decline in performance.

**Fig 3:**
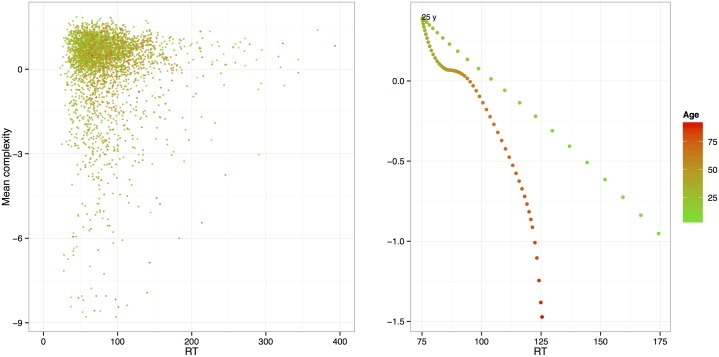
Scatterplot and developmental change trend of the CT and complexity combined. The trend is obtained by use of smooth splines of CT and complexity (df = 7).

Plotting complexity and CT trends on a single two-dimensional diagram allowed a finer representation of these developmental changes (Fig 3). It confirmed the entanglement of complexity (accuracy) and CT (speed). In the first period of life (<25), accuracy and speed increased together in a linear manner. The adult years were remarkable in that complexity remained at a high level for a protracted period, in spite of a slow decrease of speed during the same period. This suggest that during the adult period, people tend to invest more and more computational time to achieve a stable level of output complexity. Later in life (>70), however, speed stabilizes, while complexity drops in a dramatic way.

These speed-accuracy trade-offs were evident in the adult years, including the turn toward old age. During childhood, however, no similar pattern is discernible. This suggests that aging cannot simply be considered a “regression”, and that CT and complexity provide different complementary information. This is again supported by the fact that in the 25–60 year range, where the effect of age is reduced, CT and complexity are uncorrelated (*r* = −.012, *p* = .53). These findings add to a rapidly growing literature that views RIG tasks as good measures of complex cognitive abilities [21, for a review].

We have gone further here in several respects than any previous literature. First, we present a set of data collected in RIG tasks with a broad variety of instructions as to what and how to randomize: our participants playfully solved binary randomization tasks along with multiple-alternative variants; they explicitly attempted to generate random sequences, but also distributed their responses in a guessing task, typically considered “implicit random generation”. The expected outcome was unidimensional in some tasks and two-dimensional in others; constraints imposed by working memory capacity were high in some tasks, but almost absent in others. In the cognitive science literature, such diverse tasks have never been compared directly. We do not deny that the various tasks we used may tap into slightly different subcategories of prefrontal functioning, with some relying more on working memory and others on inhibitory control. Yet, we set out to illustrate the commonalities among the different tasks leaving a more fine-grained analysis to future studies.

Cross-sectional studies should try to relate behavioural complexity to the degree of maturation or degeneration of specific prefrontal cortex regions. Neuropsychological investigations could use the tasks and measures employed here with selected patient groups to perform lesion-symptom mappings, as has been done recently [57], but preferably in patients with focal vascular lesions. In parallel with such investigations, Internet-based work such as the project presented here may still play a powerful role. They may complement RIG tasks with brief behavioural tasks having a known neurocognitive basis and well-studied developmental trajectories. Thus, laboratory testing and web-based approaches may conjointly help pinpoint the cognitive mechanisms underlying the age-related modulation of behavioural complexity.

A second extension of the existing literature on subject-generated random sequences is the number of participants tested and their age-range. To date, only two studies have investigated age-related changes in RIG tasks with a range comparable to the one investigated here [15, 22]. They both compared groups of young adults and older adults and were thus unable to describe the continuous evolution of complexity across the lifespan.

Finally, one of the most exciting novel aspects of this research is that we have presented an estimate of algorithmic complexity that relies on sequences shorter than any that research on RIG reported in the psychological literature would have dared to use because of the limitations of other complexity indexes.

### Conclusion

RIG tasks require a sense of randomness or complexity, as well as cognitive functions such as attention, inhibition and working memory. The evolution of algorithmic complexity over the lifespan is compatible with the idea that RIG tasks, even in a computerized and shortened format, reflect such abilities. The developmental curve reveals an evolution compatible with the concept of a combination of fluid and crystallized components in cognition, with the latter predominating.

Beyond the similarity of complexity trajectories, we found that the variety of RIG tasks offered different and probably complementary information about a participant’s cognitive abilities. The exact component of cognition that is assessed by RIG tasks, and which factors differentiate the tasks, are still open questions.

Our findings shed light on the developmental change in ASC, on which inductive reasoning about randomness is built. They will hopefully further our understanding of human probabilistic core knowledge. Like other complex cognitive abilities, the trend in evidence here must not occlude important intra- and inter-subject variations. Age (squared) explains about 2% of the variance in mean complexity, and 4% of the variance in CT. Although age is the predominant variable, CT and complexity are also affected, in the case of some tasks, by sex, statistical intuition and paranormal belief. Future research should investigate the impact of other variables on RIG performance. Examples comprise a participant’s tendency to persevering which would have to be established in an independent task. Alternatively, use of computers and familiarity with an online environment might be considered.

Anonymized data are available from https://github.com/algorithmicnaturelab/HumanBehavioralComplexity
